# Farther Observations and Facts Relative to the Practice of Inoculation of the Small Pox

**Published:** 1787

**Authors:** John Covey

**Affiliations:** Apothecary at Basingstoke, in Hampshire


### LONDON MEDICAL JOURNAL,

FOR THE YEAR 1787,

### PART THE FIRST.

I. *Farther Obſervations and Facts relative to the Practice of Inoculation of the Small Pox. Communicated in a Letter to Dr.* Simmons, *F.R.S. by Mr.* John Covey, *Apothecary at Baſingſtoke, in Hampſhire.*

*To Dr.* SIMMONS.

SIR,

IF you think the following obſervations merit a place in the Medical Journal, you will oblige me by inſerting them, in addition to thoſe ſome time ſince ſent * by,

SIR,

Your moſt obedient ſervant,

JOHN COVEY.

*Baſingſtoke,*
Nov. 27th, 1786.

* Vol. VII. page 180.

 CRUDE

CRUDE matter, taken from the inoculated part in its early ftage, although it may generally prove efficacious, and is ftrongly recommended by feveral refpectable writers, is not always to be depended upon.

Mr. Mudge fays, "This mode of proceed-
"ing, he has great reafon to believe, is a very
"uncertain method of infecting the patient.
"Poifoning the fkin or part itfelf to which
"the virus is applied, locally, and infecting
"the conftitution generally, are two diftinct
"confiderations. The fimple inflammation of
"the puncture, and the fubfequent puftular
"appearance, being no proofs that the poifon
"has reached the conftitution. That Meffrs.
"Langworthy and Arfcott, furgeons, inocu-
"lated thirty patients with matter taken in its
"crude ftate from the arm of a young woman,
"five days after fhe herfelf had been inocu-
"lated; that although the infection took place
"on their arms, producing a large prominent
"puftule, with matter in it, yet not one of
"them had the fmall pox in confequence of
"it*." And Baron Dimfdale, on this fact, obferves, "that the failure moft probably arofe

* Mudge, on Inoculation, from page 17 to page 23.

"from

" from want of confidering accurately, whe-
" ther fuch inflammation and fore were truly
" of a variolous nature, which, in the prefent
" inftance, he thought was not the cafe; and
" that the thirty perfons were inoculated from
" a difcharge not impregnated with the vario-
" lous poifon *."

I was myfelf fome time paft informed of a family that were inoculated at a houfe fet apart for this purpofe, who, after a flight degree of illnefs, and a few eruptions of fhort duration, were fent home in thirteen or fourteen days, as having paffed in that expeditious manner through the real fmall pox; but the event left no room to doubt of their having been inoculated with matter from a fpurious pox, they all being taken ill foon after their return, and having the true difeafe in the natural way.

On the 20th of laft month I inoculated nine perfons with frefh crude matter, taken on a thread from the arm of a young man, on the tenth day from his inoculation, and before he had any eruption. The arms of two of thefe began to inflame, and the fmall pox appeared at the ufual periods. The incifions on the

* Dimfdale's Tracts on Inoculation, page 188.

other other ſeven began to inflame the day after they were made; on the fourth, the inflammation was ſo greatly increaſed, and moſt of them diſcharged ſo conſiderably, as to have afforded matter ſufficient to have inoculated fifty perſons; on the fifth and ſixth, the greater part of them were covered with large prominent ſcabs, the inflammation being much abated; on the eighth, many of the ſcabs were rubbed off, and the inflammation was ſo far gone, as to make it doubtful whether the matter had taken a proper effect or not; on the ninth, two of them began complaining, as did three of the others the three following days, the inciſions being again ſomewhat inflamed, with a ſlight degree of pain in the axilla. On the other two of theſe ſeven, the ſcabs being gone, and no ſign of inflammation remaining, I inoculated them again, on the tenth day from the firſt inoculation, with concocted matter; theſe inciſions began to inflame the third day thereafter, and gradually increaſed till the fifth; the patients then ſickened, and the eruption appeared on the ſeventh. They all had the diſtemper in the mildeſt manner.

Notwithſtanding theſe facts ſpeak greatly in favour of concocted matter; yet I would by no means be underftood as affirming, or even believing, that the fame effects will always follow the ufe of crude matter; moft certain, however, it is, the progrefs was in thefe inftances very irregular: poffibly, the early and great difcharge from the incifions might be the caufe that feveral of thefe patients fickened fo much later than ufual, and that in two of them the variolous infection appeared to be entirely carried off.

Neither is the thin watery ichor, contained in the large vefications which fometimes appear in the fmall pox, proper for the purpofe of inoculation; of this I have known fome inftances, at one time on three patients, at another time on four; the fame being introduced each time in its fluid ftate by puncture, and yet failing in its effect.

Matter taken on a thread from the faireft puftules, a day or two before they begin to turn off, carefully dried and kept from the damp air, anfwers every intention of the inoculator, when placed in a flight incifion, and retained therein for a fhort time by a roller without plafter; with thefe additional advantages, that no miftake can eafily be made in the diftemper by the operator; the patient may reft reſt ſatisfied, that he is ſecure from future infection, and at the ſame time ſuch accidents as are related by Meſſrs. Frewen, Mudge, and Quier, will be avoided.

Mr. Frewen ſays, that "Mr. Dutton, ſurgeon, informed him, that he unwarily gave a woman the ſmall pox by bleeding her with a lancet, which he had uſed nine days before in opening ſome puſtules, in order to take matter for inoculation *."

Mr. Mudge gives a ſimilar inſtance †:

And Mr. Quier ſays, "he can by no means recommend the uſe of a lancet with matter dried on it; the wounds with which the ſame are made moſt commonly inflaming as much the day after they are made, as they uſually do the fifth day, when freſh fluid matter is uſed; that about the third day this inflammation begins to ſubſide, and then gradually inflames again, provided the infection ſucceeds; that by ſuffering the matter to dry on the lancet, it will be ſo blunted, as to require more force to be uſed in making the inciſion; the conſequence of which

* Frewen's Practice and Theory of Inoculation, page 25.

† Mudge, on Inoculation, page 13.

" will be, a ſpeedy inflammation and digeſ-

" tion of the wound, and by the diſcharge

" the variolous infection will ſometimes be

" carried off:" and of this he gives two inſtances*.

And, indeed, when any conſiderable numbers are to be inoculated, and there is no variolous patient near, the uſe of dried thread will, I think, be far preferable to the mode of infecting a number of lancets ſufficient for the purpoſe, or drying matter, and then moiſtening it with the ſteam of hot water, or diluting it with a ſmall proportion of the ſame.

Dr. Schultz, who for a conſiderable time attended the hoſpital for inoculation in London, ſays, " The thread wherewith the operation of
" inoculation is performed, is to be prepared
" in the following manner: the tops of a few
" mature puſtules are opened with a lancet on
" the arms or legs, and a cotton thread of the
" neceſſary length is moiſtened with the puru-
" lent matter. This thread is then gently
" dried by the fire, and afterwards laid up in
" a wooden box. Some make uſe of a cloſe

* Eſſays and Letters on the Small Pox, Inoculation, &c. page 72.

"ſtopped glaſs; but that is not ſo eligible, "for Mr. Wall, ſurgeon to the Small Pox "and Chriſt hoſpitals, once perceived a pu- "trid ſmell out of the glaſs, perhaps, becauſe "the thread had not been ſufficiently dried." And that Dr. Fothergill told him "of ſuch "another inſtance; and obſerved, that if the "thread is not dried, the matter is much more "ſubject to putrefaction *."

Dr. Wall ſays, "that where the thread is to "be kept long before it is uſed, it may be beſt "to dry it a little before it is ſecured in the "phial from the external air, as by this means "any putrefactive fermentation in the pus "may be prevented. He had been told, that "animalcula had been obſerved in the thread, "when put in wet, and kept a conſiderable "time †."

I have myſelf formerly ſeen ſome inſtances, where cotton, imbued with variolous matter, and put wet into a glaſs cloſely ſtopped, has ſoon grown putrid and mouldy.

I acknowledge, I could never diſcover any advantages attending punctures, and the uſe of

* Schultz, on Inoculation, page 47.

† Andrew, on Inoculation, page 52.

fluid fluid matter, ſuperior to thoſe attending ſlight inciſions, and the ſame matter dried, provided it had not been long taken before it was uſed; in this latter caſe, the effects are ſaid to be, and, I believe, are, ſlower; and it has been apprehended, the diſtemper has ſometimes proved leſs favourable.

Dr. Monro ſays, " Five perſons were inocu-" lated with long-kept matter; on the four-" teenth day thereafter, one of theſe fevered, " and in two or three days more the ſmall pox " appeared. This patient recovered. Three " others fevered ſix weeks after the inocula-" tion; one of theſe recovered, the others " died. The fifth had no ſmall pox at all*."

Although, as before obſerved, I have myſelf never had occaſion to make uſe of any variolous matter that had been taken more than ſix weeks, having ever made choice of it as freſh as poſſible, yet I am in no doubt of its keeping a much longer time: but how long, is a circumſtance, I believe, not hitherto determined, and whilſt freſh matter is to be procured, is an inquiry ſavouring more of curioſity than utility; poſſibly, ſo long as the thread

* Monro, on Inoculation in Scotland, page 36.

retains its ſpringineſs, it will prove efficacious.

Dr. Kirkpatrick relates a caſe where ſome was uſed, ſeemingly with ſucceſs, which had been taken almoſt ſix years: but when it is conſidered, that the thread was here applied in the manner of a ſeton, and moved about each day; that the patient ſickened the ſixth; that the eruption appeared the eighth; and that the natural diſeaſe had been in the ſame houſe a ſhort time previous to the uſe of it; there is certainly much reaſon to doubt whether the ſmall pox was received from the operation or not; and that the Doctor himſelf had his ſcruples, I think, appears probable from his own relation of the caſe: moſt certain it is, little, if any thing, could be learnt from the appearance of the punctured part*.

In addition to what was ſaid in my laſt, relating to the inoculated ſmall pox anticipating the natural infection, I have farther to obſerve, that, for many years paſt, on finding one in a family ill with the ſaid diſorder, and where the eruption has not been out more than three,

* Kirkpatrick's Analyſis of Inoculation. Second edition, Page 212.

four, four, five, or fix days, I have advifed thofe in the fame family who had not already had it, to be immediately inoculated, and I have very rarely met with an inftance of its having deceived my expectations.

As a ftrong confirmation that the inoculated fmall pox does fuperfede the natural infection, I fhall relate the following recent fact.

On the 4th of Auguft laft, I admitted Elizabeth Head into my inoculating houfe, fhe having a full crop of the coherent fmall pox, which began coming out upon her the 1ft, in the forenoon; fhe had with her a child about four months old, which had been conftantly fuckled by her from the time fhe was firft taken ill, and continued fo to be during the whole of her illnefs. I inoculated this child with matter taken from its mother foon after their admiffion into my houfe; the punctured part inflamed properly; on the 11th, the child was fretful and feverifh, and on the 14th, a very favourable eruption appeared, and the child was from this period free from complaint.

Sir George Baker gives two fimilar inftances, the one from his own knowledge, the other from the relation of Dr. Watfon, of fucking children inoculated with fuccefs the fourth or fifth day after the ſmall pox had appeared on their mothers*.

Theſe are convincing proofs, how little foundation there is for the dread of accumulating infection, either by this or any other means, ſo much attended to by many practitioners.

I apprehend that a rigid preparation, together with repeated mercurial medicines, expoſure to the cold air, &c. has proved injurious to many tender conſtitutions.

Sir George Baker ſays, " that a general pre-

" parative method, although it has been at-

" tended with more ſucceſs than could reaſon-

" ably have been expected, has not always

" been practiſed with impunity; that perſons

" of tender habits have ſuffered greatly from

" the repeated uſe of ſtrong purgatives, added

" to a ſevere courſe of abſtinence; and that in

" a few inſtances of children, convulſions, and

" even death itſelf, have been the conſequence

" of this treatment: ſuch, indeed, are the ef-

" fects likely to be produced in this practice,

" whenever the diſeaſe is preſcribed to, and

" not the patient." He obſerves farther, that

* Baker, on Inoculation. Medical Tranſactions. Vol. II. Page 310.

" various

"various are the ill effects which have been

"produced by the indiscrete use of purga-

"tives, given with a view to suppress and repel

"the eruption. The most common, as well

"as the most fatal, is a consumption of the

"lungs. The late Dr. Blanshard informed

"him, that out of ninety persons who had

"been inoculated in one village, ten died con-

"sumptive, soon after their recovery from the

"small pox; three only of which number had

"shewn any disposition to diseased lungs be-

"fore their inoculation; that all these peo-

"ple had taken strong purgative medicines

"through every stage of the disease*."

Dr. Glass says, "that an imperfect crisis will

"account for the languishing and consumptive

"cases, which too often, if common report

"says true, follow the present very cooling

"process. All the evils, however, produced

"by the modern method, seem to be owing to

"the empirical practice of carrying it to the

"same length in all cases, and in most cases to

"a much greater length than is necessary or

"proper †."

* Baker, on Inoculation. Med. Transf. Vol. II. page 301.

† Glass's Second Letter to Dr. Baker, on Inoculation, page 47.

And

And Dr. Makittrick Adair has given feveral cafes of the fatal effects attending the repellent plan, which he fays, "ought to be, in general, "more moderately ufed, and in fome cafes to- "tally deferted*."

Upon the whole, I am in fome doubt whether it is not equally as fafe to follow the advice of Dr. Gatti †, and inoculate without any preparation at all, except in fome particular habits, which may evidently require it.

Dr. Frafer, of Antigua, fays, "It is, in- "deed, difficult to conceive what preparation "can be preferable to found health, or what "can prove a more proper nidus for the re- "ception of the variolous matter, than a body "whofe juices are mild and balfamic, without "acrimony of any kind, and fuch as circulate "freely, without exceeding in proportion to "the veffels which contain them. It follows "from this, that thefe previous courfes ought "to vary in different fubjects; and that it is "little lefs than empiricifm to prefcribe with-

* Adair, on the ill Effects of the repellent plan. Medical Commentaries. Vol. VIII. page 211.

† Gatti's Obfervations on Inoculation. Tranflated by Maty.

"out

" out diſtinction, what is only adapted to a

" particular caſe*."

Since my laſt, I have obſerved two caſes ſimilar to thoſe related by Dr. Monro, where the inciſions made on the arms of two children out of three ſhewed no ſigns of inflammation, and yet they both ſickened on the eighth day from the inoculation, the ſmall pox ſoon appearing on them of a bad ſort, and they both died: but as the other child's arm inflamed, and diſcharged matter in eight days, and then healed, and ſhe did not ſicken till the twenty-fourth or twenty-fifth day after being inoculated, it is reaſonable to ſuppoſe the matter made uſe of had loſt its efficacy, and that they all took the infection in the natural way; the ſmall pox of a bad kind raging in the ſame place, twelve other children dying of it within the ſame week, and in the ſame ſtreet †.

I have likewiſe, ſince that time, on looking over Mr. Quier's account of inoculation at Jamaica, obſerved, that matter taken on the eighth day from the inciſion made upon a

* Letters and Eſſays on the Small Pox, Inoculation, &c. Page 108.

† Monro, on Inoculation in Scotland. Page 36.

negro woman who had already had the diſeaſe, communicated the true ſmall pox to three negro boys, into whoſe arms the ſame was infuſed. From whence he concludes, that the variolous ferment, being introduced by an inciſion into the ſkin of a perſon who has already had the ſmall pox, and conſequently is no longer capable of being infected with that diſorder, may raiſe a local inflammation, and aſſimilate the juices contained in the part into its own nature, ſo as to enable the pus formed of them, when infuſed into another body which has never undergone the ſmall pox, to excite in it that ſpecific diſeaſe *.

And Dr. Ruſh, of Philadelphia, ſays, that Dr. Way, of Wilmington, in Newcaſtle county, informed him, that he made a puncture in his own arm with a lancet dipped in variolous matter; that notwithſtanding he had already had the ſmall pox, the ſpot where the puncture was made inflamed, and in the uſual number of days was filled with matter; that he inoculated a patient with ſome of this mat-

* Letters and Eſſays on the Small Pox, Inoculation, &c. page 100.

ter; that the patient took the infection in the usual time, and had the fmall pox in confequence thereof *.

Thefe facts are ftrong evidences in favour of the obfervations on the fame fubject by Mr. Dawfon †; but whether the application of fuch matter will be conftantly, or even generally, attended with the fame effect, future experience muft determine.

A cafe having lately fallen under my care, which appeared to me to be a convincing proof of the fuperior efficacy of bark and acids in that moft dreadful illnefs, the bloody fmall pox, which, as Sydenham obferves, "Peftem "ipfam pernicie æquabat;" and is almoft conftantly "Mortis prænunciæ." — I beg leave to relate it.

Mafter Carver, aged twelve years, naturally of a weak and tender habit of body, was removed in the morning of the 28th of July laft, from a neighbouring boarding fchool, to my inoculating houfe. He had been taken ill the 23d; the next day he had complained of violent pain in his head and back, and was at times delirious;

* Medical Obfervations and Inquiries, Vol. V. page 40.

† See Vol. VII. page 66.

 thefe these complaints rather increafed the 25th, and on the evening of the 26th were attended with fome eruptions, which were difcovered to be the fmall pox in the night of the 27th. I firft faw him foon after his arrival at my houfe: his face and hands were covered with the confluent kind; his body and other parts were likewife much loaded, running together in clufters in many places: there were a confiderable number of black fpots on his body, hips, and thighs, but more efpecially in the middle of thefe clufters. He had an extremely quick pulfe, a hot and dry fkin, a parched tongue; complained of pain in his head, back, loins, and ftomach; breathed with fome degree of difficulty, and was fomewhat delirious. He had juft made nearly a pint of urine, greatly loaded with blood. As I was informed he had had no ftool for the two or three preceding days, I gave him an opening medicine, which, in the courfe of the day, produced two motions, likewife much loaded with blood, and in the enfuing night one other, more confiderable in quantity, and more deeply coloured with the fame; I likewife gave him every three or four hours, a draught, ex decoct. cortic. Peruvian rub. fort. ℥ifs. tinct. ejufd. fp. ʒifs. c.

ſpir. vitrioli ten. gut. xx., and diluted with aq. hordeat. c. gum arabici, in each draught of which ten drops of the ſame ſpirit were infuſed. The morning of the 29th I found him cooler and more compoſed, the oppreſſion on his breath gone, and the delirium having left him ſoon after the laſt ſtool; he perſevered in the uſe of the draughts, drops, &c., the whole of this day, throughout which his water was nevertheleſs as bad as it was the preceding day. On the 30th he had another ſtool, leſs loaded with blood, but his urine was the ſame; the medicines, &c. were repeated. The 31ſt he had another motion, free from blood, and the water was leſs tinged with it. On the 1ſt of Auguſt he appeared much better, his urine was of a natural colour, and the feveriſh heat gone; but he was greatly reduced: the drops were now omitted, and the draughts, made more cordial, were repeated; proper nouriſhment being ſtrictly attended to. In this ſtate he continued until the 5th, when, nature being nearly exhauſted, he grew inſenſible at times, and departed in the enſuing night.

Now, although this caſe ended fatally, yet I think the good effects of the medicines were very conſpicuous; for notwithſtanding both his urine urine and ftools contained fo large a proportion of blood, as in part to coagulate when cold, yet a ftop was put to this moft alarming fymptom; and notwithftanding his great weaknefs, the puftules (on his face more particularly) advanced much better than could have been expected; the black fpots being likewife almoft gone, and I entertained fome hopes of his recovery till the morning preceding his diffolution. As no attention had been paid to the colour of his water, it is uncertain whether he had voided any blood with it before his removal or not.

I have very frequently experienced the good effects of the bark in almoft every kind of bad fmall pox, often beginning the ufe of it foon after the eruption, and perfevering with it throughout the illnefs, not neglecting, however, to add to it fuch other medicines as from time to time feemed neceffary; but during, as well as previous to, the exhibition of this medicine, (when given in fubftance more particularly) a ftrict attention is to be paid to the ftate of the bowels, as coftivenefs, which frequently attends the ufe of it, will, in great meafure, deftroy its good effects.

Much

Much pains have been taken by Dr. Haygarth * to fet in a true point of view feveral particulars relating to infection. As the two following cafes are on the fame fubject, I beg leave to fubjoin them :

Two brothers had the fmall pox in the worft degree; under the fame roof lived a man, his wife, his mother, and four children. Some time after the death of one of the young men, and the recovery of the other, the old woman was taken ill with the ufual fymptoms preceding the fame diftemper; the family being poor, no medical affiftance was called in, and the neighbours being fearful of the fmall pox, refrained from vifiting them. On the fifth day of her illnefs the fick perfon died, and it was with fome difficulty two women were procured to lay out the body. No eruptions were vifible on it, excepting a number of fmall purple fpots. About fourteen days thereafter the whole of this family, the two women who had affifted, the fon of one of them, and a girl who had wantonly attended at the grave, were taken ill, and all had the fmall pox. The boy

* Haygarth's Inquiry how to prevent the Small Pox, page 48.

moſt probably took the infection from his mother on her return home, the girl from the dead body at the grave, they living at a conſiderable diſtance from the infected houſe, and not having been near it.

The two women who attended Maſter Carver, (whoſe caſe is juſt related) prior to his removal from ſchool, were taken with the ſmall pox in the courſe of a fortnight, notwithſtanding he was removed within forty hours after the firſt appearance of the eruption, and both died of it.

Although theſe facts militate againſt Dr. Heberden's obſervation *, yet I have experienced a great number of caſes in confirmation of the truth of his remark. As there muſt, therefore, ever be ſo great uncertainty whether the infection is received or not, the propriety of an immediate inoculation, under ſuch circumſtances, is evident, as the moſt probable means of avoiding the ill conſequences that may, and frequently do, follow.

* Haygarth's Inquiry how to prevent the Small Pox, page 51, and London Medical Journal, Vol. VI. page 209.

II. *Some*

