# Errata

**Published:** 1787

**Authors:** 


					Page 17, Ot the prefent Volume, for cafes Jtmilar to thofe related, read
cajes related

				

